# Widespread *Treponema pallidum* Infection in Nonhuman Primates, Tanzania

**DOI:** 10.3201/eid2406.180037

**Published:** 2018-06

**Authors:** Idrissa S. Chuma, Emmanuel K. Batamuzi, D. Anthony Collins, Robert D. Fyumagwa, Luisa K. Hallmaier-Wacker, Rudovick R. Kazwala, Julius D. Keyyu, Inyasi A. Lejora, Iddi F. Lipende, Simone Lüert, Filipa M.D. Paciência, Alexander Piel, Fiona A. Stewart, Dietmar Zinner, Christian Roos, Sascha Knauf

**Affiliations:** Sokoine University of Agriculture, Morogoro, Tanzania (I.S. Chuma, E.K. Batamuzi, R.R. Kazwala, I.F. Lipende);; German Primate Center, Leibniz-Institute for Primate Research, Göttingen, Germany (I.S. Chuma, L.K. Hallmaier-Wacker, S. Lüert, F.M.D. Paciência, D. Zinner, C. Roos, S. Knauf);; Jane Goodall Institute, Kigoma, Tanzania (D.A. Collins);; Tanzania Wildlife Research Institute, Arusha, Tanzania (R.D. Fyumagwa, J.D. Keyyu);; Tanzania National Parks, Arusha (I.A. Lejora);; Liverpool John Moores University, Liverpool, UK, and Greater Mahale Ecosystem Research and Conservation Project, Kigoma (A. Piel, F.A. Stewart)

**Keywords:** spirochetes, nonhuman primates, Africa, yaws, eradication, Treponema pallidum, bacteria, infection, One Health, Tanzania

## Abstract

We investigated *Treponema pallidum* infection in 8 nonhuman primate species (289 animals) in Tanzania during 2015–2017. We used a serologic treponemal test to detect antibodies against the bacterium. Infection was further confirmed from tissue samples of skin-ulcerated animals by 3 independent PCRs (*polA*, *tp47*, and *TP_0619*). Our findings indicate that *T. pallidum* infection is geographically widespread in Tanzania and occurs in several species (olive baboons, yellow baboons, vervet monkeys, and blue monkeys). We found the bacterium at 11 of 14 investigated geographic locations. Anogenital ulceration was the most common clinical manifestation; orofacial lesions also were observed. Molecular data show that nonhuman primates in Tanzania are most likely infected with *T. pallidum* subsp. *pertenue*–like strains, which could have implications for human yaws eradication.

The geographic distribution of infection with the bacterium *Treponema pallidum* in nonhuman primates (NHPs) in Africa has been reported to closely match the one seen in human yaws in Africa before the first yaws eradication campaign (*1*). Some Africa countries, such as Tanzania, have a history of human yaws but lack recent epidemiologic data that support elimination (*2*). At the same time, many of these countries report NHP infection with *T. pallidum* strains that are highly similar to the human yaws–causing *T. pallidum* subsp. *pertenue* (TPE) (*3**,**4*; S. Knauf et al., unpub. data, https://www.biorxiv.org/content/early/2017/05/10/135491) and thus make NHP infection an important issue for a One Health approach.

The first published report of *T. pallidum* infection in Tanzanian NHPs came from anogenital ulcerated olive baboons (*Papio anubis*) at Gombe National Park (GNP) in the late 1980s (*5*), followed by cases reported from olive baboons at Lake Manyara National Park (LMNP) (*3*,*6*,*7*) and Serengeti National Park (SNP) (*3*). Clinical manifestations of *T. pallidum* infection in NHPs ranged from asymptomatic to severe skin ulceration mainly affecting the face or genitalia (*8*). Although early serologic investigations conducted by Fribourg-Blanc in West Africa confirmed widespread infection in several NHP species (e.g., baboons [*Papio* sp.], guenons [*Cercopithecus* sp.], red colobus [*Piliocolobus badius*], and chimpanzees [*Pan trogoldytes*]) (*9*), the infection in Tanzania was exclusively reported from olive baboons in northern parts of the country. Despite the close genetic relationship to human yaws–causing TPE strains (*3**,**4*; S. Knauf et al., unpub. data, https://www.biorxiv.org/content/early/2017/05/10/135491), and in the absence of recent reports of human yaws in Tanzania (*10*), it is currently unclear whether NHP strains naturally infect humans.

As a starting point and basis for advanced epidemiologic investigations, our main objective was to investigate the geographic distribution and host species composition of *T. pallidum* infection in free-ranging NHPs in Tanzania. We hypothesized that, in Tanzania, A) NHPs other than olive baboons are infected with the *T. pallidum* bacterium and B) that infection is not restricted to northern parts of the country.

## Materials and Methods

### Study Design, Sampling Sites, and Animals

**Figure 1:**
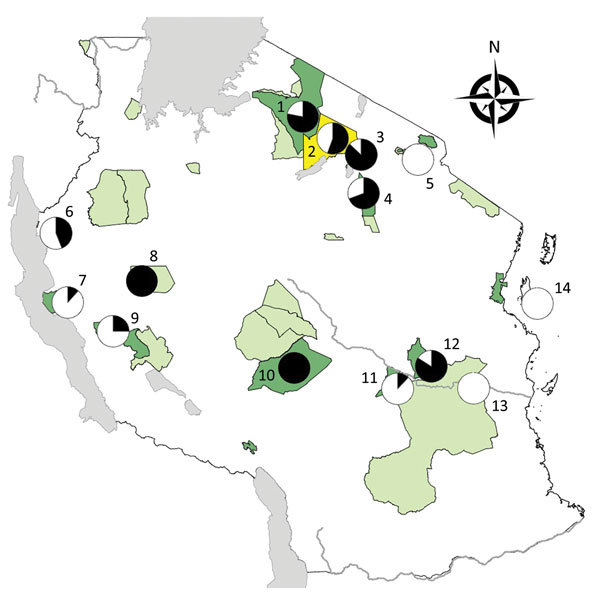
Protected areas and sites where free-ranging nonhuman primates (NHPs) were sampled in a study of *Treponema pallidum* infection, Tanzania. 1, Serengeti National Park (41 NHPs); 2, Ngorongoro Conservation Area (18 NHPs) 3, Lake Manyara National Park (38 NHPs); 4, Tarangire National Park (26 NHPs); 5, Arusha National Park (14 NHPs); 6, Gombe National Park (32 NHPs); 7, Mahale National Park (17 NHPs); 8, Issa Valley (2 NHPs); 9, Katavi National Park (12 NHPs); 10, Ruaha National Park (18 NHPs); 11, Udzungwa National Park (25 NHPs); 12, Mikumi National Park (25 NHPs); 13, Selous Game Reserve (8 NHPs); 14, Jozani-Chwaka Bay National Park–Masingini Forest on Unguja Island, Zanzibar (13 NHPs). Dark green indicates national parks; light green indicates game reserves; yellow indicates conservation area. Circle graphs: black, NHPs *T. pallidum*–positive (serology and/or PCR); white, NHPs *T. pallidum*–negative (serology and PCR). The map was produced with ArcMap version 10.0 (ESRI, Redlands, CA, USA) by using shape files available from ESRI (national boundary of Tanzania, water bodies of Africa, main rivers of Africa). The shape files of the conservation areas of Tanzania were provided by the Tanzania National Park Authority and are available free from http://www.arcgis.com/home/item.html?id = 9b06fe723ad14991b30b1b85953224c1. Prevalence circles were generated using Excel version 15.38 (Microsoft, Redmond, WA, USA).

We applied a cross-sectional study design using semirandom selection of free-ranging NHPs in selected areas in Tanzania. Selection of NHPs was biased toward animals with visible skin ulcers. Sampling took place at Arusha National Park (ANP), GNP, Katavi NP (KNP), LMNP, Mahale NP (MNP), Mikumi NP (MKNP), Ngorongoro Conservation Area (NCA), Ruaha NP (RNP), Selous Game Reserve (SGR), SNP, Tarangire NP (TNP), Udzungwa NP (UNP), and Issa Valley (Issa), as well as Jozani-Chwaka Bay NP−Masingini Forest (JCBNP) on Unguja Island, Zanzibar (Figure 1). We investigated the following species: olive baboon, yellow baboon (*Papio cynocephalus*), blue monkey (*Cercopithecus mitis*), red-tailed monkey (*Cercopithecus ascanius*), vervet monkey (*Chlorocebus pygerythrus*), Udzungwa red colobus (*Piliocolobus gordonorum*), Zanzibar red colobus (*Piliocolobus kirkii*), and Ugandan red colobus (*Piliocolobus tephrosceles*). Using FreeCalc (http://epitools.ausvet.com.au/content.php?page=FreeCalc2), and based on our previous study at LMNP (*6*) that showed a disease prevalence of 85%, we calculated a sample size of >4 (expected disease prevalence 85%) to 21 (expected disease prevalence 25%) per sample site as statistically sufficient to demonstrate freedom from *T. pallidum* infection using imperfect tests and allowing for small populations (Technical Appendix 1).

### Anesthesia and Sampling

We studied the animals in accordance with applicable regulations and guidelines (Technical Appendix 1). The sampling of blood and skin tissue followed a standardized protocol that we previously applied for baboons (*6*,*11*). In brief, the NHPs were chemically immobilized by remote distance injection of 10.0 mg ketamine/kg body mass (Kyron Laboratories, Johannesburg, South Africa) in combination with 0.2 mg/kg medetomidine (Domitor; Pfizer, Berlin, Germany). Anesthetics were intramuscularly injected using a cold-gas immobilization rifle (MOD JM, Dan-Inject ApS, Børkop, Denmark) and appropriate projectiles. Immobilized NHPs were continuously observed for vital parameters such as respirations, pulse frequency, and internal body temperature. We monitored pulse frequency and blood oxygen saturation using a Nellcor OxiMax N65 Pulse Oximeter (Tyco Healthcare Deutschland GmbH, Neustadt, Germany). Anesthetized animals underwent a standardized health check with special focus on skin lesions. We collected whole blood from the femoral vein using an S-Monovette closed blood collection system (Sarstedt, Nümbrecht, Germany) mounted with a 20G needle. We collected two 9-mL serum tubes under aseptic conditions. We then centrifuged serum tubes at 55,000 relative centrifugation force for 15 min, transferred serum into cryovials, and stored the vials in liquid nitrogen. In animals with skin lesions, we took a 6-mm biopsy from the skin ulcer using a sterile dermal biopsy punch. From each animal (and ulcer), we preserved tissue samples in lysis buffer (10 mmol/L Tris [pH 8.0], 0.1 EDTA, and 0.5% sodium dodecyl sulfate).

We treated animal wounds with Silverspray (Silver Aluminum Aerosol; Henry Schein, Langen, Germany) and allowed animals to recover under close supervision. Samples were temporarily stored at −80°C at the Tanzania Wildlife Research Institute headquarters (Arusha, Tanzania). Aliquots were exported to the German Primate Center (Göttingen, Germany) for further analysis and additional confirmation.

### Serologic Testing

We used a commercially available treponemal test (ESPLINE TP, Fujirebio Diagnostics, Hannover, Germany) to check all serum samples for *T. pallidum* antibodies. The assay has been validated for use in baboons (*12*), where it performed with 97.7% (95% CI 87.7%–99.9%) sensitivity and 96.0% (95% CI 79.7%–99.9%) specificity. We tested serum samples on the day of sampling and operated and interpreted test cassettes according to the manufacturer’s guidance.

### DNA Extraction from Skin Tissue

We extracted DNA following the standard protocol of the QIAmp DNA Mini Kit (QIAGEN, Hilden, Germany), with some minor modifications. In brief, we cut ≈25 mg tissue into small pieces and incubated in 180 μL lysis buffer, in which the sample had been stored since collection. After adding 20 μL proteinase K, samples were digested overnight at 56°C and 900 rpm (Thermomixer Comfort; Eppendorf, Hamburg, Germany). We added an additional washing step using 300 μL AW1 buffer and eluted the DNA twice with 100 μL AE buffer. We further purified extracted DNA using glycogen precipitation according to the protocol published in Knauf et al. (*13*).

### *TP_0105* (*polA*) Amplification and Sequencing

We performed PCR targeting the polymerase 1 gene (*TP_0105*, *polA*) of *T. pallidum* by using primers designed by Liu et al. (*14*). This assay has a reported sensitivity of 95.8% and a specificity of 95.7% and has been demonstrated to segregate pathogenic *T. pallidum* subspecies from nonpathogenic treponemes, other spirochetes, and 59 species of bacteria and viruses including those causing genital ulcers in humans (*14*). The 50-μL reaction volume comprised 25 μL 2 × Universe High-Fidelity Hot Start DNA Polymerase Master Mix (Biotool, Munich, Germany), 17 μL RNAase free water, 2 μL of each 10 μmol/L primer, 1 μL DNA polymerase (1 U/μL), 1 μL of 10 mmol/L each dNTP, and 2 μL template DNA, independent of DNA concentration. We conducted amplification in a SensoQuest Labcycler using the following thermocycling conditions: predenaturation at 95°C for 3 min, followed by 50 cycles each with 95°C for 15 s, 60°C for 15 s, and 72°C for 30 s. The profile was completed with a postextension step at 72°C for 5 min and indefinite cooling of the PCR product at 8°C. All *polA* PCR products were run on a 1% agarose gel to check for PCR performance and correct amplicon size. We gel extracted a representative subset of the PCR products (n = 19), purified with the QIAGEN Gel Extraction Kit (QIAGEN), and Sanger sequenced using the BigDye Terminator Cycle Sequencing Kit (Applied Biosystems, Foster City, CA, USA) and the amplification primers. Sequencing was performed by Seqlab Sequence Laboratories (Microsynth, Göttingen, Germany).

### *TP_0574* (*tp47*) Quantitative PCR

We performed TaqMan real-time PCR targeting a 132-bp fragment of the *TP_0574* gene. Primers and probe used were published elsewhere (*15*). The reaction encompassed 10 μL TaqMan Universal MasterMix II (no Uracil-N glycosylase; Applied Biosystems) and 1.8 μL of each 10 μmol/L primer and the probe. Total genomic DNA concentration added to each reaction was normalized to 100 ng. Molecular-grade water was used to adjust the reaction volume to 20 μL. Cycling conditions were as follows: 50°C for 2 min, 95°C for 10 min, followed by 50 cycles each at 95°C for 15 s and 60°C for 60 s. Reactions were run on a StepOnePlus Real-Time PCR System (Applied Biosystems). We measured all samples as triplicates and analyzed data using StepOne version 2.3 software (Applied Biosystems).

### *TP_0619* Amplification and Sequencing

We performed PCR targeting the *TP_0619* gene of *T. pallidum* to distinguish infection with TPE or *T. pallidum* subsp. *endemicum* (TEN) strains from infection with *T. pallidum* subsp. *pallidum* (TPA) strains. At this locus, TPA differs from TPE and TEN in >73 positions (Technical Appendix 1 Figure). We used primers 5′-TTACCCAGACATTTTTCTCCACATA-3′ and 5′-TACAAGCTCCCACAATGCCA-3′ to amplify a 608-bp fragment. The PCR conditions and working steps were identical to the PCR targeting the *polA* gene, except that the annealing temperature was adjusted to 55°C.

### Data Analysis

We performed statistical analyses using GraphPad Prism version 7.0c (GraphPad Software, La Jolla, CA, USA), and R version 3.3.2 (R Foundation for Statistical Computing, Vienna, Austria). We compared variables such as the presence of *T. pallidum* antibodies and clinical manifestations per species by using 2 × 2 × *n* contingency tables and a 2-tailed Fisher exact test. We used a χ^2^ test to compare the outcome of >2 sampling sites using *n* × 2 contingency tables. Proportions were tested at a critical probability of 0.05 and 95% CI. We considered p<0.05 as statistically significant.

We analyzed and edited retrieved sequence data using 4Peaks 1.8 (http://www.nucleobytes.com) and SeaView 4.5.4 software (*16*). We compared sequences with respective orthologs available in GenBank using a standard nucleotide BLAST search (http://blast.ncbi.nlm.nih.gov/Blast.cgi).

## Results

### NHP Species

We sampled 289 NHPs (Table) and confirmed previously reported *T. pallidum* infection in olive baboons at GNP (*5*,*17*,*18*), SNP (*3*), NCA (*3*), and LMNP (*3*,*6*,*7*). In addition, we report *T. pallidum* infection in yellow baboons, vervet monkeys, and blue monkeys in different regions of Tanzania (Table; Figure 1; Technical Appendix 2).

**Table:** Test results of *Treponema pallidum* infection in samples of free-ranging nonhuman primate species, Tanzania*

Species	No. (%)	Total/seropositive/skin lesion/PCR positive†						
		Positive				Negative		
		Total	Male	Female		Total	Male	Female
Olive baboon (*Papio anubis*)	137 (47.4)	86	34/34/12/12	52/51/31/30‡		51	29/0/1‡/NA	22/0/1‡/NA
Yellow baboon (*Papio cynocephalus*)	75 (26.0)	33	17/17/2/2	16/16/5/5		42	27/0/1§/NA	15/0/0/NA
Vervet monkey (*Chlorocebus pygerythrus*)	45 (15.6)	35	21/21/10/8‡	14/14/1/1		10	7/0/0/NA	3/0/0/NA
Blue monkey (*Cercopithecus mitis*)	15 (5.2)	2	1/0/1/1	1/1/0/0		13	8/0/0/NA	5/0/0/NA
Red-tailed monkey (*Cercopithecus ascanius*)	2 (0.7)	0	NA	NA		2	2/0/0/NA	NA
Zanzibar red colobus (*Piliocolobus kirkii*)	10 (3.5)	0	NA	NA		10	4/0/0/NA	6/0/0/NA
Udzungwa red colobus (*Piliocolobus gordonorum*)	3 (1.0)	0	NA	NA		3	2/0/0/NA	1/0/0/NA
Ugandan red colobus (*Piliocolobus tephrosceles*)	2 (0.7)	0	0/0/0/0	0/0/0/0		2	2/0/0/NA	NA
Total	289 (100.0)	156	73	83		133	81	52

*Results are based on the consensus of detected *T. pallidum* antibodies (ESPLINE TP) and PCR results of 3 independent gene targets (*polA*, *tp47*, and *TP_0619*). NA, not applicable. †PCR was conducted only on animals with skin lesions. ‡No skin sample was available for some positive animals. §Skin lesion at the genitalia most likely from fight; no tissue sample available.

The overall mean seropositivity of *T. pallidum* infection in the NHP samples was 53.3% (154/289). More female (82/135 [60.7%]) than male (72/154 [46.8%]) NHPs had *T. pallidum* antibodies. Overall, 35/45 (77.8%) vervet monkeys, 85/137 (62.0%) olive baboons, 33/75 (44.0%) yellow baboons and 1/15 (6.7%) blue monkeys had antibodies against the bacterium. Most (94 [61.0%]) of the 154 seropositive NHPs appeared healthy without any clinical skin lesions. The association between *T. pallidum* antibodies and skin ulceration was tested using 2-tailed Fisher exact test and was significant in olive baboons (n = 137; odds ratio [OR] 15.95 [95% CI 4.7–51.1]; p<0.0001) and yellow baboons (n = 75; OR 11.04 [95% CI 1.7–126.8]; p = 0.0185), but not in vervet (n = 45; OR ∞ [95% CI 0.0–1.0]; p = 0.0888) and blue monkeys (n = 15; OR 0.00 [95% CI 0.0–126.0]; p>0.9999 [dataset is provided in Technical Appendix 2]). No *T. pallidum* antibodies were detected in the 10 Zanzibar red colobus, 3 Udzungwa red colobus, 2 Ugandan red colobus, and 2 red-tailed monkeys sampled. Moreover, none of these 4 species showed any kind of skin ulceration (Table).

### Clinical Manifestations

**Figure 2:**
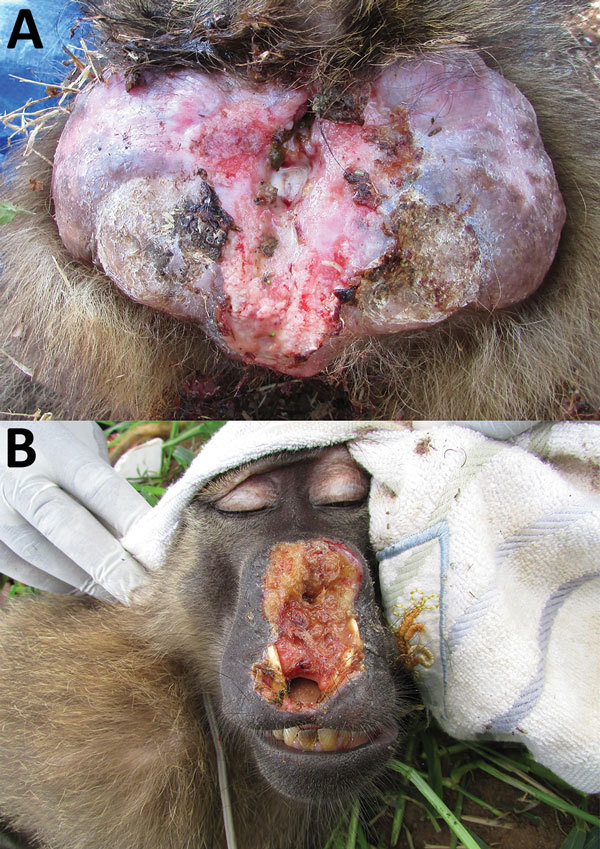
*Treponema pallidum*–induced clinical manifestations affecting olive baboons (*Papio anubis*), Tanzania. A). Lesions on the anogenital area of animal at Lake Manyara National Park. B) Facial lesions of animal at Tarangire National Park. Orofacial lesions were found only in olive baboons.

Among the 156 *T. pallidum*–seropositive and/or PCR-positive NHPs (including 2 serologically negative but PCR-positive animals) and across the different sampling sites, we found anogenital ulcers associated with the infection (Figure 2, panel A) in 59.8% ± 23.9% of the yellow baboons (mean ± SEM, 6 investigated sites; data were analyzed as fraction of *T. pallidum*–infected animals with anogenital lesions per sampling site); 45.6% ± 16.2% of the olive baboons (mean ± SEM, 6 investigated sites); and 31.6% ± 9.4% of the infected vervet monkeys (mean ± SEM, 9 investigated sites). One of the 2 *T. pallidum*–infected blue monkeys showed anogenital skin ulceration; the second animal was clinically healthy. Orofacial lesions (Figure 2, panel B) were exclusively observed in olive baboons at SNP, TNP, and LMNP, of which 2 olive baboons at TNP and 1 at SNP were included in our study. These animals represent 3.5% of the 86 *T. pallidum* –seropositive and/or PCR-positive sampled olive baboons. One animal from TNP had concurrent orofacial and anogenital skin ulcerations. We also observed these ulcerations in olive baboons at LMNP, although capture and sampling of these animals was not possible.

### Geographic Distribution

Our results provide evidence for *T. pallidum* infection in NHPs at 11 of the 14 sites investigated (Figure 1; Technical Appendix 2). The only sites where infection was not detected were ANP (14 NHPs), SGR (9 NHPs), and JCBNP (13 NHPs). We found *T. pallidum*–positive vervet monkeys in all areas where the species was examined (GNP, KNP, LMNP, MKNP, MNP, RNP, SNP, TNP, UNP) except for the 1 animal from Zanzibar (JCBNP). One PCR-positive and anogenital ulcerated blue monkey from LMNP had reproducibly negative serologic results. Because sampling was biased toward animals with skin lesions, we more objectively compared field sites by analyzing data from animals that appeared to be clinically unaffected. Healthy-looking olive baboons were significantly more often *T. pallidum*–positive at LMNP (n = 6/6) than at any other sampling area in Tanzania where the species is present (ANP [n = 0/12], GNP [n = 8/23], NCA [1/9], SNP [n = 16/25], TNP [n = 12/17]; 6 × 2 contingency table: χ^2^ = 30.15, df = 5; p<0.0001). Likewise, clinically unaffected yellow baboons were significantly more often *T. pallidum*–infected at MKNP (n = 16/19) than at any of the other sampling areas in Tanzania where the species is present (KNP [n = 0/6], MNP [n = 0/10], RNP [n = 8/16], SGR [n = 0/7], UNP [n = 2/17]; 6 × 2 contingency table: χ^2^ = 38.39, df = 5; p<0.0001). In the vervet monkeys, we found no differences among sampling sites (GNP [n = 3/3], KNP [n = 2/5], LMNP [n = 1/2], MKNP [n = 2/3], MNP [n = 1/2], RNP [n = 4/4], SNP [n = 8/8], TNP [n = 3/6], JCBNP [n = 0/1]; 9 × 2 contingency table: χ^2^ = 12.97, df = 8; p = 0.1130), but sample size per site was low (Technical Appendix 2).

### Molecular Characterization of *T. pallidum* Samples

In the 65 animals with skin ulcers, we confirmed *T. pallidum* by amplification of a part of the *polA* gene (classic PCR) and/or the *tp47* locus (quantitative PCR; 59/60 animals tested positive; Technical Appendix 2). For 5 animals, we did not perform PCR because of limited quantities of samples. All obtained sequences were identical. We deposited a representative sequence of the *polA* gene from a yellow baboon (16RUF8140716) in GenBank (accession no. MF627733). Of 58 tested animals, 56 were positive in the PCR targeting the *TP_0619* locus. For 7 NHPs, no PCR was performed because of sample limitations. Again, all 41 sequences obtained were identical. We deposited a representative sequence from a vervet monkey (4KNF2121016) in GenBank (accession no. MF754122). The haplotype was identical to those derived from TPE and TEN strains but different from TPA strains in >73 positions (Technical Appendix 1 Figure).

## Discussion

We confirmed *T. pallidum* infection in 4 free-ranging NHP species at 11 of 14 investigated sites in Tanzania. Our data for GNP must be interpreted with caution. GNP has a history of treating infected baboons with antimicrobial drugs (*17*), which might have affected prevalence rates and clinical manifestations. The finding that clinically unaffected olive baboons at LMNP, but also many animals at SNP and TNP, were infected with the bacterium (as indicated by serology; Table) shows that clinical manifestations are not representative of the actual prevalence of the disease. This finding is consistent with reports from an earlier investigation of olive baboons at LMNP in 2007 (*6*) and in Guinea baboons (*Papio papio*) in the Niokolo Koba National Park, Senegal (*11*). In the context of human *T. pallidum* infection, where a latent stage is a key feature of infection (*19*) and which equally features positive serology in the absence of active skin lesions (*20*), this finding could argue for a similarity of disease progression in the NHP host. However, in the absence of long-term monitoring data for infected NHPs, relapsing cases, which would indicate the latent stage, cannot be identified, and standardized laboratory infection might be needed to obtain those data.

Although reduced susceptibility for *T. pallidum* infection is possible in some of the investigated species (colobines), it is likely that infection is not yet present because of behavioral and ecologic constraints between the infected and noninfected species. At least in a recent publication, a Ugandan red colobus was described with suspected active yaws-like lesions in Uganda (*21*). Consequently, we note that our sample size for colobines and red-tailed monkeys was insufficient. As a result, a conclusive evaluation on possible *T. pallidum* infection in these species was not possible. The same applied for sites where the number of infected NHPs was critically low, for example, UNP and MNP or the negative tested areas at ANP (14 animals), SGR (9 animals), and JCBNP (13 animals), as well as the NCA crater region where all 8 olive baboons were tested negative. We found *T. pallidum*–infected vervet monkeys with and without skin ulcers in 9 of the 10 sites where the species has been investigated. This finding and the larger number and geographic extent of *T. pallidum* infection in *Chlorocebus* sp. (*4*,*11*,*22*–*24*) deserve further attention in prospective studies, especially in areas where the species is present but has not yet been tested.

All *T. pallidum*–positive NHPs in this study revealed a *TP_0619* sequence that points toward infection with either TPE or TEN strains (Technical Appendix 1 Figure). In the context of the geographic distribution of TEN strains (dry areas in Sahelian Africa and western Asia) (*25*) as well as the information obtained from the whole-genome sequences of the Tanzanian simian strains LMNP1 and 2 (S. Knauf et al., unpub. data, https://www.biorxiv.org/content/early/2017/05/10/135491), which are considered TPE strains, we assume that TPE is the dominant, if not exclusive, *T. pallidum* subspecies infecting Tanzanian NHPs. Further clarification will be achieved when multilocus strain typing data and whole-genome sequence data of the NHP samples become available.

In humans, TPE is mainly transmitted by direct skin-to-skin contact (*26*). A possible important alternate route of infection has been discussed through the involvement of flies as a vector (*27*,*28*). Although both options are at least theoretically possible for NHPs (*13*), direct contact should be considered the most likely way of intraspecies and interspecies transmission. Such transmission is further supported by reports of the close association and interaction (play, fight, or hunt) among different NHP species (*29*–*31*). Again, multilocus strain typing and whole-genome sequence data of the strains infecting NHPs in Tanzania are likely to contribute to a better understanding of host–pathogen coevolution and will provide details of the relatedness of the *T. pallidum* subspecies that infect the different NHP taxa.

Human yaws is known to be endemic to 13 countries, but Tanzania is among the 76 countries with a known history of the disease that lack recent epidemiologic data (*2*). More precisely, the disease was reported to be endemic in humans in the western areas along Lake Tanganyika and in southern Tanzania (*32*). Extensive elimination efforts decreased the reported incidence of human yaws in Tanzania from 120,000 cases in 1927 to 52,000 in 1950 (*33*) and 71 in 1978 (https://web.gideononline.com). At the same time, the wide distribution of *T. pallidum* infection in NHP on Tanzania’s mainland (*7*) and the chronic infection with locally high prevalence rates (e.g., LMNP [*6*]) suggest the pathogen has been present in the respective NHP populations for at least several decades. However, current data are insufficient to develop a conclusive biogeographic scenario about the origin and spread of the infection. The first published report on *T. pallidum* infection in NHPs in 1989 (*5*) involved olive baboons at GNP. Although this is no evidence for the origin of *T. pallidum* infection in NHPs in Tanzania, it is interesting in the context of a possible anthropozoonotic introduction of the disease. GNP is in the region that has been historically classified as an area to which human yaws in Tanzania is endemic (*33*). Furthermore, GNP is close to the Democratic Republic of the Congo, a country that still reports cases of human yaws (*34*). However, all of this is speculative, and whole-genome data are needed from NHPs and human strains from the same area to provide a deeper understanding on the origin and transmission of *T. pallidum* in NHPs in Tanzania.

In a larger context, neighboring countries currently do not report NHPs with *T. pallidum*–confirmed skin lesions, although animals from East Africa (not further classified) (*22*) and Kenya (*3*) have tested serologically positive. Because *T. pallidum* infection in NHPs in Africa is widespread (*1*), further investigations should specifically include more East Africa countries, particularly those that share their borders with Tanzania.

We showed that *T. pallidum* infection in NHPs in Tanzania is geographically widespread and present in several Old World monkey species, namely olive and yellow baboons, vervet monkeys, and blue monkeys (hypothesis A). We identified the pathogen in almost all investigated sites covering large parts of Tanzania’s mainland (hypothesis B) and showed that NHPs in Tanzania are most likely infected by TPE strains. Nevertheless, our overall sample size does not permit a conclusive statement on *T. pallidum* prevalence in NHPs at any of the sampled sites. Further studies on the spatial distribution of NHP infection with *T. pallidum* and advanced genetic characterization of simian strains are crucial for identifying NHPs as a possible reservoir for human infection (*35*). In light of the data and for a sustainable eradication of human yaws, a One Health approach in which animal and human health is investigated (*36*) is needed.

Technical Appendix 1 Ethics statement, sample size calculation and interpretation, and alignment of the *TP_0619* sequence data.

Technical Appendix 2 Dataset of all free-ranging nonhuman primates included into the study.
