# Supplementary material for: Widespread Treponema pallidum Infection in Nonhuman Primates, Tanzania
Source: Emerg Infect Dis. 2018 Jun;24(6):1002–9. doi: 10.3201/eid2406.180037 (PMC6004850; doi:10.3201/eid2406.180037)
Supplement: Technical Appendix 1 — Ethics statement, sample size calculation and interpretation, and alignment of the TP_0619 sequence data. [file 18-0037-Techapp-s1.pdf]

Article DOI: https://doi.org/10.3201/eid2406.180037

Widespread *Treponema pallidum* Infection in Nonhuman Primates, Tanzania

Technical Appendix 1

Sample Size Calculation

We used FreeCalc, a calculator for sample size for freedom testing with imperfect test available through http://epitools.ausvet.com.au/content.php?page=FreeCalc2. The tool is based on the methods published by Cameron and Baldock in 1998 (*1*) and is used to calculate the required sample size and cut point for testing to demonstrate population freedom from disease using imperfect tests and allowing for small populations. Two assumptions were tested. First, we used the disease prevalence known from baboons at Lake Manyara National Park (*2*) and second, we tested for the scenario with a much lower disease prevalence (25%) (Technical Appendix 1 Table).

Interpretation

If a random sample of 4 units is taken from a population of 1,000 and $\leq$1 reactors are found, the probability that the population is diseased at a prevalence of 0.85 is 0.0145.

If a random sample of 21 units is taken from a population of 1,000 and $\leq$2 reactors are found, the probability that the population is diseased at a prevalence of 0.25 is 0.0444.

Ethics Statement

Free-ranging nonhuman primates (NHPs) were chemically immobilized and sampled in accordance with the requirements of the relevant guidelines and regulations, in particular the Tanzania Veterinary Act No. Sixteen of 2003 and Tanzania Wildlife Research Institute’s (TAWIRI) Guidelines for Conducting Wildlife Research (2012; http://tawiri.or.tz/wp-content/uploads/2017/05/Wildlife-research-guideline.pdf). Respective permits for wildlife-

protected areas were issued by the Commission for Science and Technology in Tanzania (2015–89-NA-2014–228), Ministry for Natural Resources and Tourism (Wildlife Division, HA.403/563/01B/90, 178/606/01/115 and HA.178/606/01/6), Tanzania National Parks (TNP/HQ/C.10/13), and Ngorongoro Conservation Area Authority (NCAA/D/240/Vol.XXV/130) as well as the Revolutionary Government of Zanzibar through the second Vice-President's Office (Zanzibar Research Committee OMPR/M.95/C.6/2/Vol.IV/60). The study methods including the animal handling protocols were reviewed and approved by the Animal Welfare and Ethics Committee of the German Primate Center (E10–17) and the Vice Chancellor of Sokoine University of Agriculture (SUA/ADM/R.1/8). We applied "Good Veterinary Practice" rules to all procedures where animals were handled. Registered veterinarians immobilized NHPs and closely monitored anesthetized animals until they fully recovered.

References

1. Cameron AR, Baldock FC. A new probability formula for surveys to substantiate freedom from disease. Prev Vet Med. 1998;34:1–17. PubMed http://dx.doi.org/10.1016/S0167-5877(97)00081-0

2. Knauf S, Batamuzi EK, Mlengeya T, Kilewo M, Lejora IA, Nordhoff M, et al. *Treponema* infection associated with genital ulceration in wild baboons. Vet Pathol. 2012;49:292–303. PubMed http://dx.doi.org/10.1177/0300985811402839

3. Knauf S, Dahlmann F, Batamuzi EK, Frischmann S, Liu H. Validation of serological tests for the detection of antibodies against Treponema pallidum in nonhuman primates. PLoS Negl Trop Dis. 2015;9:e0003637. [Erratum in: PLoS Negl Trop Dis. 2015;9:e0003757]. PubMed http://dx.doi.org/10.1371/journal.pntd.0003637

Technical Appendix 1 Table. Sample size calculations of free-ranging nonhuman primates included in the study of *Treponema pallidum* Infection, Tanzania.

Variable and results	*T. pallidum* prevalence 85%	*T. pallidum* prevalence 25%
Input variable		
Test sensitivity*	0.98	0.98
Test specificity*	0.96	0.96
Population size	1,000	1,000
Design prevalence	0.85	0.25
Diseased elements	850	250
Analysis method	Modified hypergeometric exact	Modified hypergeometric exact
Target Type I error	0.05	0.05
Target Type II error	0.05	0.05
Population threshold for infinite probability formula	10,000	10,000
Maximum sample size	100	100
Results		
Required sample size	4	21
Cut-point number of positives	1	2
Type I error	0.0145	0.0444
Type II error	0.0091	0.0497
Population-level sensitivity	0.9855	0.9556
Population-level specificity	0.9909	0.9503

*Espline TP (*3*).

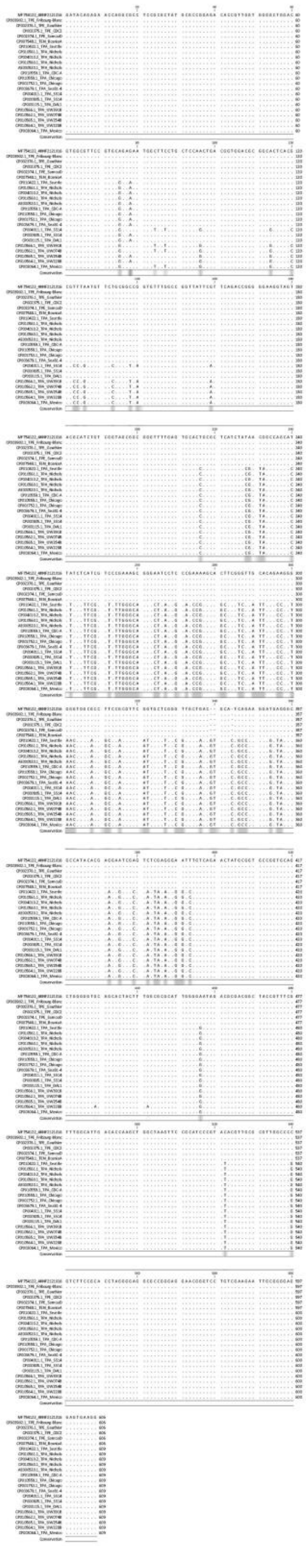

Technical Appendix 1 Figure. Alignment of the *TP_0619* sequence data.
